# A Practical Electronic Health Record-Based Dry Weight Supervision Model for Hemodialysis Patients

**DOI:** 10.1109/JTEHM.2019.2948604

**Published:** 2019-10-24

**Authors:** Zhaori Bi, Mengjing Wang, Li Ni, Guoxin Ye, Dian Zhou, Changhao Yan, Xuan Zeng, Jing Chen

**Affiliations:** 1 National Clinical Research Center for Aging and Medicine, Huashan HospitalFudan University 12478 Shanghai200040China; 2 Division of Nephrology, Huashan HospitalFudan University 12478 Shanghai200040China; 3 Department of Electrical EngineeringThe University of Texas at Dallas 12335 RichardsonTX75080USA; 4 State Key Laboratory of ASIC & SystemDepartment of MicroelectronicsFudan University 12478 Shanghai200433China

**Keywords:** Personalized prognosis, personalized risk prediction, electronic health record, hemodialysis

## Abstract

*Objective*: Dry Weight (DW) is a typical hemodialysis (HD) prescription for End-Stage Renal Disease (ESRD) patients. However, an accurate DW assessment is difficult due to the complication of body components and individual variations. Our objective is to model a clinically practicable DW estimator. *Method*: We proposed a time series-based regression method to evaluate the weight fluctuation of HD patients according to Electronic Health Record (EHR). A total of 34 patients with 5100 HD sessions data were selected and partitioned into three groups; in HD-stabilized, HD-intolerant, and near-death. Each group’s most recent 150 HD sessions data were adopted to evaluate the proposed model. *Results*: Within a 0.5 kg absolute error margin, our model achieved 95.44%, 91.95%, and 83.12% post-dialysis weight prediction accuracies for the HD-stabilized, HD-intolerant, and near-death groups, respectively. Within a 1%relative error margin, the proposed method achieved 97.99%, 95.36%, and 66.38% accuracies. For HD-stabilized patients, the Mean Absolute Error (MAE) of the proposed method was 0.17 kg ± 0.04 kg. In the model comparison experiment, the performance test showed that the quality of the proposed model was superior to those of the state-of-the-art models. *Conclusion*: The outcome of this research indicates that the proposed model could potentially automate the clinical weight management for HD patients. *Clinical Impact*: This work can aid physicians to monitor and estimate DW. It can also be a health risk indicator for HD patients.

## Graphical abstract

This paper presents a clinically practicable dry weight estimator for hemodialysis (HD). The proposed time series-based regression method to evaluate the weight fluctuation of HD patients achieved achieved 95.44%, 91.95%, and 83.12% post-dialysis weight prediction accuracies for HD-stabilized, HD-intolerant, and near-death groups, respectively (0.5 kg absolute error margin). The proposed model could potentially automate clinical weight management for HD patients.

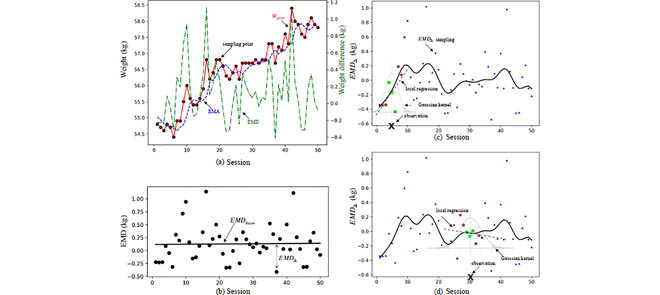

## I. Introduction

Chronic Kidney Disease (CKD) characterized by the gradual loss of kidney function is commonly recognized as one of the most severe global public health problems. CKD affected more than 753 million people and caused 1.2 million deaths in the year of 2016 [1]. The disease is the 18-th leading cause of death in globally [2]. The end-stage of CKD is called End-Stage Renal Disease (ESRD), and patients with ESRD must take Renal Replacement Therapy (RRT) including dialysis and transplantation. As of 2010, the number of individuals receiving RRT worldwide was reported as 2.6 million, with over two-thirds of them relying on Hemodialysis (HD) treatment. By the year of 2030, the number is expected to double roughly [3]. Although developing countries provide the most market for HD consumptions, the medical conditions and quality of HD there are limited. The availability of experienced clinicians and well-conditioned HD centers [4] can hardly meet patients’ demands. The urge to expand high-quality HD services has been brought to attention.

The primary objective of HD is to remove the free water that is excreted by healthy kidneys in the form of urine from the blood via Ultrafiltration (UF). To determine the UF volume for HD session, clinicians must evaluate the patients’ fluid statuses with the prescription of Dry Weight (DW), which is defined as patients’ lowest tolerated post-dialysis weight at which the patients have the minimal signs or symptoms of hypovolemia or hypervolemia [5]. Overestimation or underestimation of DW causes harmful consequences including cardiovascular disease, malnutrition, and increased rate of hospitalization and mortality [6]. Though many methods are proposed to approximate DW [7]–[10], none of them can be adopted as a golden standard or has shown better survival benefits [11]. The difficulty in estimating DW stems from variations and uncertainties of the human system, such as nutrition status, underlying illness, commodities, etc. Thus, the trial-and-error method is still widely applied in clinical situations. Experienced clinicians still estimate DW with the closest accuracy to the truth, but it requires an incredible amount of time and effort. We aim to develop personalized DW estimation models and implement automatic DW evaluation applications to supervise HD patients’ weight adjustment behaviors. The proposed models could enhance the quality of HD and help to fulfill the enormous demands of high-quality dialysis services. The following segment contains a brief overview of the relevant works.

### A. Related Work

Time series modeling aims to establish the connections between observed data and future events. It fits excellently with demands on medical applications where patient records often correlated with time and observatory markers. Many studies have focused on time series model developments [12], [13]. We summarized the conventional methods into two categories: (1) statistics-based techniques—works under this class usually elaborate models with problem-dependent assumptions, such as linearity, periodicity, data distributions, the order of the model, and more. Autoregressive Integrated Moving Average (ARIMA) is a representative model in a stochastic time series analysis [13]. According to different applications, it derives various subclass models, such as Autoregressive (AR), Moving Average (MA), Autoregressive Moving Average (ARMA), and Seasonal ARIMA (SARIMA) [14]–[16]. Though there are limitations for statistics-based methods, they have still proven capable of achieving remarkable performances with specific problem. (2) Machine learning based methods — works in this category have high feasibility to solve real-world problems with the characteristics of non-linearity, minimal knowledge of priori distributions, high dimensional variables. Artificial Neural Network (ANN) [17] is a a typical scheme that can approach the complex system with rigorous precision. There are also many variations of ANN-based methodologies, such as Long Short Term Memory (LSTM) network [18], Time Lagged Neural Network [19], and other implementations [20]. We also noticed that Random Forest (RF) [21], Support Vector Machines (SVM) [22], and Bayesian Networks (BN) [23] are wildly applied in literature because of their steady performance. Worth mentioning is that machine learning schemes face the difficulties in parameter optimizations, over-fitting functions, and model interpretability. Though many techniques have been proposed to provide solutions to these problems, some questions remained to be solved.

Studies exist on the use of machine learning assisted HD applications. For example, in an HD anemia treatment, the ANN method [24] was utilized to determine hemoglobin levels in HD patients, and [25] a reinforcement learning method to optimize the dose of erythropoiesis-stimulating agents has also been proposed. The RF model [26] has been applied to predict HD patients’ cardiovascular risk, and a decision tree method [27] to detect early Arteriovenous Fistula (AVF) Failure has been adopted. In regards to HD quality control, a Temporal Abstractions (TA) method [28] to monitor the quality of HD process has been proposed, and an applied Bayesian network [29] to recognize patient temporal-state transition patterns and detect the exception events is in place. A study [30] proposed a Bioimpedance analysis (BIA) based on a multiple variable regression model to predict DW, and the accuracy is controlled within 0.5 kg with a standard deviation of 2 kg. Research [31] has applied the Multi-Layer Perceptron (MLP) neural network to predict DW using the inputs of patients’ BIA and blood volume monitoring data, with findings revealing a 0.5 kg outcome with a standard deviation of 1.3 kg. Although studies [30], [31] have reported significant DW modeling progress, the model remains dependent on crowd data, which may lead to data bias. Moreover, we propose that DW is a dynamic value, and a personalized training model is necessary for achieving the better precision. The studies listed here illustrate the remarkable potential of the application of machine learning methodology in HD.

The rest of the paper is organized as follows: Section II explains the proposed methodology, Section III shows the experimental results and comparisons with other methods, and Section IV is a detailed discussion based on the observed results. The final part is the conclusion.

## II. Methods

### A. Method Overview

Theoretically, precise DW can hardly be obtained. We treated the trial-and-error post-dialysis body weight as the domain knowledge to approximate DW value. It is worth mentioning that all the patients enrolled were received an extended period of care from medical experts, and all DW-assessed Electronic Health Record (EHR) data are from the HD center of the Huashan Hospital. The physiological variables are stored by automatic transmission from the dialysis machine or hospital information system, which makes the research data highly authentic and complete. We retrieved the HD sessions data of each HD patient and trained a customized post-dialysis weight estimation model. To explore models’ dependencies on life conditions, we partitioned patients into three groups, namely, HD-stabilized, HD-intolerant and near-death. The clinical data were collected from the devision of Nephrology, the Huashan Hospital. This research was approved by the Ethics Committee at the Huashan hospital and written informed consent was collected from all study participants.

### B. Participants

A total of 34 patients with 150 HD entries of EHR data for each were selected for the research. Participants were divided into three groups: (1) HD-stabilized group — participants in good health conditions without any sign of hypovolemia and hypervolemia or underlying illness; (2) HD-intolerant group — participants showing typical symptoms of hypotension during an HD session indicating HD-intolerant status; (3) Near-death group — participants were dead one week after the last recorded data were collected. The statistical characteristics for enrolled patients are shown in Table 1.

**TABLE 1:** Overall Statistics for the Study of Patients

Clinical Characteristic	HD-stabilized group (n=16)	HD-intolerant group (n=7)	near-death group (n=11)
Demographic characteristics			
Age (year)	57.6±10.3	71.7±11.5	73.9±7.6
Sex (male)	9(56%)	3(43%)	6(55%)
Pre-dialysis BP (mmHg)	1.4±164 \ 75.2±9.3	1.8±376 \ 63.2±14.8	1.4±227 \ 71.9±13.4
Post-dialysis BP (mmHg)	1.3±176 \ 76.7±10.1	1.5±313 \ 61.2±14.5	1.7±204 \ 68.1±10.7
Pre-dialysis weight (kg)	61±10.1	64.1±16.2	60±11
Post-dialysis weight (kg)	58.5±9.8	61.6±16.3	57.5±11.4
Dialysis vintage (year)	10.7±6.7	9.1±5.2	7.6±5.2
Etiology of end-stage renal disease			
Diabetes mellitus	2(13%)	2(29%)	4(36%)
Hypertensive nephropathy	3(19%)	3(43%)	3(27%)
Chronic Glomerulonephritis	7(44%)	0(0%)	2(18%)
Obstructive nephropathy	1(6%)	0(0%)	0(0%)
Other	3(18%)	2(28%)	2(18%)

^1^ ± means standard deviation value.

^2^ Parentheses shows percentages of patients.

^3^ \ separates systolic and diastolic blood pressure

This study aimed to establish a DW supervision system to monitor the weight adjustment behavior of HD-stabilized patients. It is a necessary measure to setup control groups to observe evidence of inaccurate predictions that may refer to exceptional medical events. Therefore, HD-intolerant and near-death groups were put in place. Details on the results from different groups are discussed in later sections.

### C. EHR Data Format

All data were auto-collected from the dialysis machine and hospital information system. Each HD session contains the following data entries: (1) Patient ID, which is a unique code for patient identity; (2) Date of HD; (3) Pre-dialysis and post-dialysis Blood Pressure (BP); (4) Pre-dialysis and post-dialysis body weight. (5) UF volume, which is the amount of fluid removed from HD patients. In this study, we excluded the BP data since they are open variables results from factors, such as patient mind status, medications, food, and others.

### D. Missing Data Treatment

We found the original EHR data missing a few weight values. The data frame was discarded if both pre-dialysis and post-dialysis weights were missing. Otherwise, the missing weights were estimated from the equation (1).

\begin{equation*} w_{pre}=w_{post}+u/1000+\delta\tag{1}\end{equation*}

where $w_{pre}$ is pre-dialysis weight, $w_{post}$ is post-dialysis weight, $u$ is the volume of UF (mL), and $\delta $ is the average weight draft which is defined by equation (2).

\begin{equation*} \delta =\frac {1}{m}\sum _{i}^{m}{\left ({w_{pre}-w_{post}-u/1000}\right)}\tag{2}\end{equation*}

Though combining the mean UF with its bias and pre-/post-dialysis weight can approximate the missing weight values. Individually, the post-dialysis weight is not simply a case of pre-dialysis weight minus UF volume (L). Figure 2 scatters the dialysis weight difference and UF volume (L). In most cases, the UF volume (converted to weight) is higher than the weight difference because of the supplementary normal saline at the end-stage of dialysis section. The implantation dose of normal saline depends on the clinical status of a patient. In a few cases the UF volume is less than the weight difference because of the excretion during the dialysis. Very few patients had residual kidney functions allowing them to urinate, causing further weight loss. Considering the above scenarios where the UF volume may indicate the dry weight status, we included the UF volume as a variable.

**FIGURE 1.**
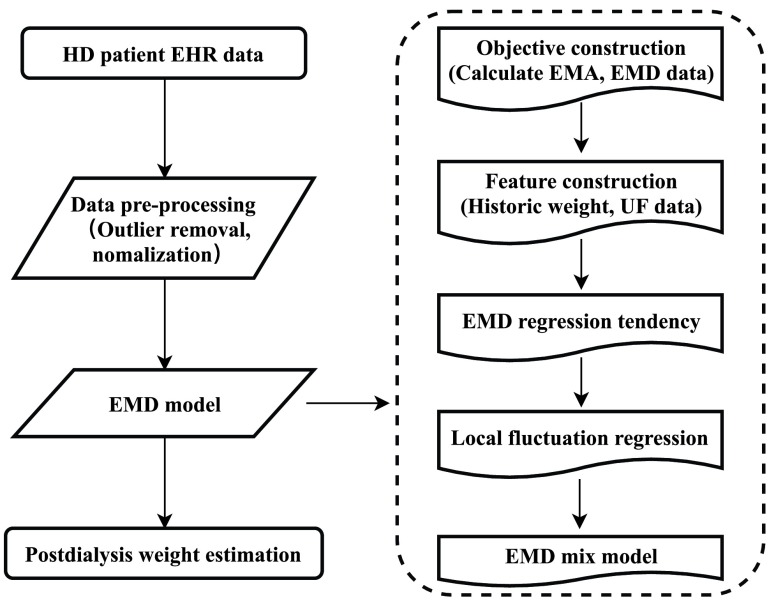
The flow diagram for $EMD$ model development.

**FIGURE 2.**
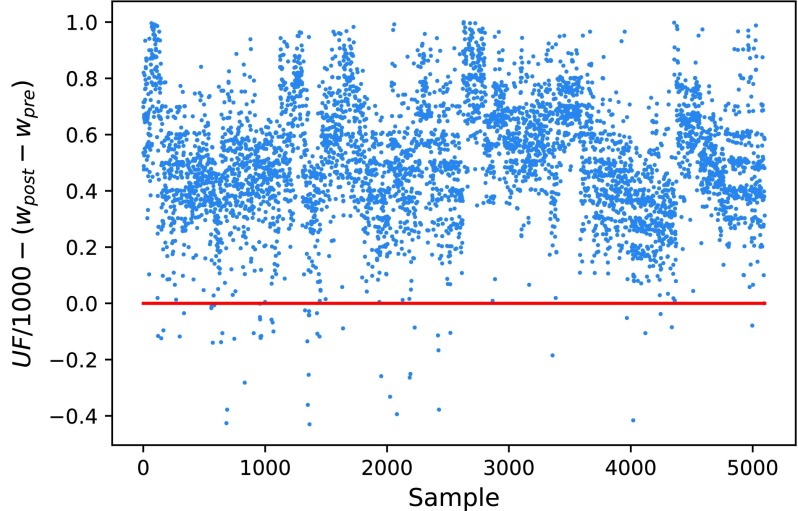
The illustration of the relationship between dialysis weight difference and UF volume. The red line is an ideal scenario where removal fluid is equal to weight loss. In most cases, the UF volume is above or lower because of the supplementary normal saline and the excretion during the dialysis.

### E. Model Features

#### 1) Historic Weight

The feature domain $F$ is defined by equation (3).

\begin{equation*} F=W_{0}^{pre}\cup W_{k}^{pre}\cup W_{k}^{post}\cup U_{k}\tag{3}\end{equation*}

where $W_{k}=\left \{{w_{1},w_{2},\ldots,w_{k}}\right \}$, $U_{k}=\left \{{u_{1},u_{2},\ldots,u_{k}}\right \}$, $k$ is the most recently k sessions measurements of weight and UF, $W_{0}^{pre}$ is the current pre-dialysis weight.

#### 2) Outlier Removal

We filtered the outlier data via the differences in pre- and post-dialysis weight values to obtain a robust mathematical model. The training set excluded the largest and the smallest weight differences in data entries with a rate of 10% for each side.

### F. Objective

#### 1) Exponential Average Difference

From clinical observations, the post-dialysis weight is time-sensitive. We used the Exponential Moving Average (EMA), which is defined by equation (4), to model the time decay effect of input features.

\begin{equation*} EMA(f)=\sum _{i=1}^{n}\alpha _{i}*f_{i}\tag{4}\end{equation*}

where $f_{i}$ is the $i$-$th$ input of the variable $f$, $\alpha _{i}$ is a time-attenuated weight factor, as shown in equation (5).

\begin{equation*} \alpha _{i}=\begin{cases} \dfrac {1}{1+\sum _{i}^{n}\left({\frac {n-1}{n+1}}\right)^{i}}*\left({\dfrac {n-1}{n+1}}\right)^{i}, & \text {if $i>1$}\\ \dfrac {n-1}{n+1}, & \text {if $i=1$} \\ \end{cases}\tag{5}\end{equation*}

The model objective, namely the $EMD$ of post-dialysis weight is defined by equation (6).

\begin{equation*} EMD(W_{post})=W_{post}-EMA(W_{post})\tag{6}\end{equation*}

$EMD$ exhibits good stationary properties whose statistical information is relatively constant over time. Thus, instead of directly connecting data features to $w_{post}$, building a model dependent on $EMD$ simplifies the future weight forecasting by utilizing system regularity. Figure 4(a) illustrates the difference between $w_{post}$, $EMA(W_{post})$, and $EMD(W_{post})$ data.

**FIGURE 3.**
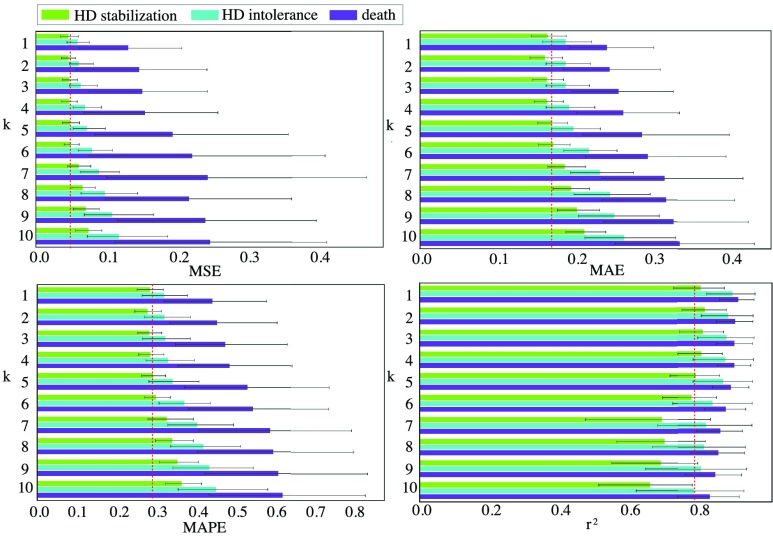
Model evaluation with the k parameter ranging from 1 to 10. For MSE, MAE, and MAPE, the lower the value, the better the performance. For $r^{2}$, the larger the value, the better the performance. The red dash line indicates k=5 performance. k is insensitive to the model when smaller than 5, and when k is larger than 5, the model accuracy degrades significantly.

**FIGURE 4.**
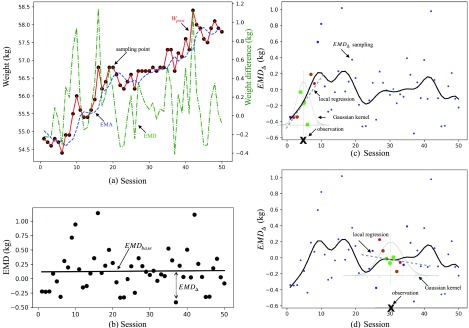
(a) A sample of the post-dialysis weight ($W_{post}$), $EMA$, and $EMD$ value curve. Left y-axis validates for $w_{post}$ and EMA, while right y-axis validates for $EMD$. The figure shows that $EMD$ is a stationary data compared with the other two weight-related variables by neutralizing the trend in raw weight and EMA data. (b) An illustration of EMD’s regression tendency. The black points are EMD samplings, the straight line $EMD_{base}$ is EMD linear regression, and the dash distance represents $EMD_{\Delta }$, which is the difference between $EMD$ and $EMD_{base}$. (c) An illustration of the local regression of $EMD_{\Delta }$. Based on the observation point, the sampling points are weighted locally through the Gaussian kernel function, as shown in equation (11). The weight is associated with the dots’ size and color, where blue dots’ weights approach to zero. (d) The same annotations with (c) but with a different observation point. Note that (c) and (d) are illustrations of local regressions for training process. The future session data will not available in real time prediction.

### G. Modeling Method

In this study, the $EMD$ value is approximated by equation (7).

\begin{equation*} \widetilde {EMD}=EMD_{base}+EMD_{\Delta }\tag{7}\end{equation*}

where $EMD_{base}$ describes the average data tendency, and $EMD_{\Delta }$ is the local correction factor. The detail implementations are discussed as the follows.

#### 1) Data Normalization

All input features are scaled to zero mean and unit variance through equation (8).

\begin{equation*} \widetilde {f_{i}}=\frac {f_{i}-Mean(f)}{STD(f)}\tag{8}\end{equation*}

where $f_{i}$ is the $i$-$th$ element of input feature $f$, $Mean()$ is the mean value function, and $STD()$ is the standard variance function.

#### 2) Linear Regression

We used the naive linear regression method to model the $EMD$ baseline ($EMD_{base}$). The formula is defined by equation (9).

\begin{equation*} EMD_{base}=\sum _{f_{i}\in F}\lambda _{i}*\widetilde {{f_{i}}}\tag{9}\end{equation*}

where $\lambda $ represents the linear regression coefficients that are solved using the least square method. An illustration of an $EMD_{base}$ modeling is shown in figure 4(b).

#### 3) Gaussian Kernel-Based Calibration

The nonlinearity of the model draft is modeled with the local weighted regression method which is defined by equation (10).

\begin{equation*} EMD_{\Delta }=EMD_{base}-EMD=\sum _{f_{i}\in F}\lambda _{i}^{\prime }*\widetilde {f_{i}}^{\prime }\tag{10}\end{equation*}

where $\widetilde {f_{i}}^{\prime }$ is weighted training data based on the current input sample $X$ and gaussian kernel method which is defined by equation (11) [32].

\begin{equation*} \widetilde {f_{i}}^{\prime }=\widetilde {f_{i}}*e^{\frac {-(f_{i}-X_{i})^{2}}{2\tau ^{2}}}\tag{11}\end{equation*}

where $\tau $ is a constant parameter. The weighted regression method can approximate the local fluctuation efficiently by biasing the Eulerian nearest training points from the history data repository. Gaussian kernel-based local regressions are illustrated in figure 4(c)-(d).

#### 4) Modeling Flow

The modeling flow is summarized in figure 1. We established the personalized post-dialysis weight model via four steps: (i) Extracting patients’ EHR data from the dialysis information system. The data of interest is defined by equation (3); (ii) Performing the data pre-processing procedure to remove outliers and fill missing values; (iii) Mixing the EMD regression tendency ($EMD_{base}$) and local regression ($EMD_{\Delta }$) results to approach the $EMD$ value; (iv) Post-dialysis weight was calculated using equation (6), where $EMA(W_{post})$ is a known variable.

### H. Method Implementations

The ARIMA method is implemented with statsmodels [33] package, the RF method is implemented with Scikit-learn [34] package, and the LSTM is implemented with TensorFlow [35] and Keras [36] packages. Figure 3 illustrates k as being insensitive to the model when smaller than 5. On the other hand, the model accuracy degrades as k increases because larger k values bring up more features that make the model harder to fit. We set the parameter $k$ in equation (3) to 5 (16 input features) for the following reasons: (1) DW is an interval value. Larger k values may give more reliable results. (2) Setting k at 5 in clinical practice can achieve a reasonable observation time window that is equivalent to 2 weeks follow-up of the patients who visit the HD center 2–3 times a week. The ARIMA model is fitted with the parameters with a lag order of 5 for autoregression, difference order is 1 for stationary data enhancement, and moving average order is set to 0. RF is set up with a maximum depth of 2 for each estimator, and the total number of estimators is 200. LSTM is constructed with one hidden layer and 16 units, and the time step is 1. The same training and testing data are fed to all methods.

### I. Performance Matrices

The model quality is evaluated via multiple measures: the Mean Absolute Error (MAE) in equation (12) that emphasizes the overall error in magnitude, the Mean Absolute Percentage Error (MAPE) in equation (13) that addresses the relative mismatch of predictions, the Mean Squared Error (MSE) in equation (14) that weights more on large deviations and the coefficient of determination $r^{2}$ that measures the fitness of regression in equation 15.

\begin{align*} MAE=&\frac {1}{n}\sum _{i}^{n}\mid EMD_{i}-\widetilde {EMD_{i}}\mid \tag{12}\\ MAPE=&\frac {1}{n}\sum _{i}^{n}\mid \frac {EMD_{i}-\widetilde {EMD_{i}}}{EMD_{i}} \mid \times 100 \tag{13}\\ MSE=&\frac {1}{n}\sum _{i}^{n}(EMD_{i}-\widetilde {EMD_{i}})^{2} \tag{14}\\ r^{2}=&1-\frac {\sum _{i=1}^{n}({EMD_{i}-\widetilde {EMD_{i}})^{2}}}{\sum _{i=1}^{n}({EMD_{i}-\overline {EMD}})^{2}}\tag{15}\end{align*}

where $\overline {EMD}$ is the mean value of $EMD$ testing samples defined in equation (16), and $n$ is the number of testing samples. A perfect predictive model occurs if $MAE=0$, $MAPE=0$, $MSE=0$, and $r^{2}=1$, otherwise, the larger the deviation, the worse the model is.

\begin{equation*} \overline {EMD}=\frac {1}{n}\sum _{i}^{n}{EMD_{i}}\tag{16}\end{equation*}

## III. Experimental Results

The proposed method was running on a 1.1 GHz Intel core M CPU with 8G 1600 MHz DDR3 memory. The average runtime of the proposed model (training and predicting for one patient) is 0.25s.

### A. Error Distributions

**FIGURE 5.**
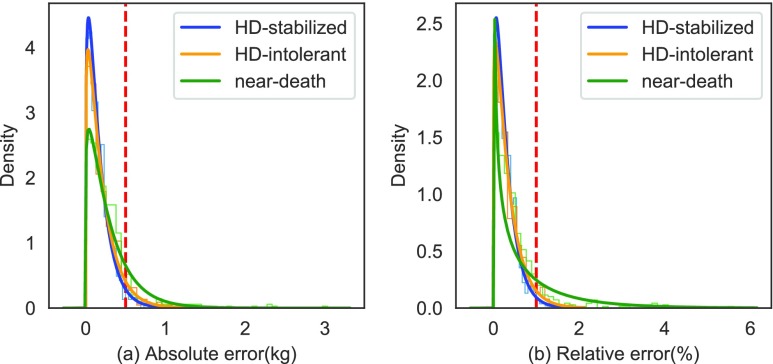
(a) The absolute error distribution of the three patients groups, where the red dash line indicates a 0.5 kg error. The CDF value with a greater than 0.5 kg error for the HD-stabilized, HD-intolerant, and near-death groups are 4.56%, 8.05%, and 16.88%, respectively. (b) The relative error distribution of the three patients groups, where the red dash line indicates a 1% error. The CDF value with a greater than 1% error for the HD-stabilized, HD-intolerant, and near-death groups are 2.01%, 4.64%, and 33.62%, respectively.

We evaluated the accuracy of the proposed method with the absolute and relative error matrices, where the absolute error is a non-negative kilogram weight difference between patients’ actual post-dialysis weights. The relative error is the absolute error that divides the patients’ post-dialysis weights. As a rule of thumb, the absolute weight error would be kept within 0.5 kg, and the relative error would be kept within 1% by the clinical staff to gain the best treatment for HD patients. We fitted the gamma error distributions in figure 5. The proposed model showed smaller variations and average errors when comparing HD-stabilized patients with patients from the other two groups. This result indicated that HD-intolerant and near-death patients’ recent historical weight data might consist of inner chaos that is challenging to model the regularity. In this sense, larger prediction errors were generated. Overall, the value of the estimated Cumulative Distribution Function (CDF) of the gamma distribution with a ≤ 0.5 kg absolute error were 95.44%, 91.95%, and 83.12% for the HD-stabilized, HD-intolerant, and near-death groups, respectively. The relative error distribution had a better performance with an error of ≤ 1% of which the CDF values were 97.99%, 95.36%, and 66.38%. From the experimental results, the accuracy of the proposed method decreases with patients’ deteriorating conditions. Patients with stable life signs (HD-stabilized) exhibited minimal DW prediction errors, indicating that our approach may be used as a novel marker to monitor HD quality. On the other hand, the proposed method can be a good fit for most HD patients, given that about 80% of patients are at stable clinical levels according to the annual data report from the United States Renal Data System (USRDS) of the year 2018 [37].

### B. Model Comparisons

**FIGURE 6.**
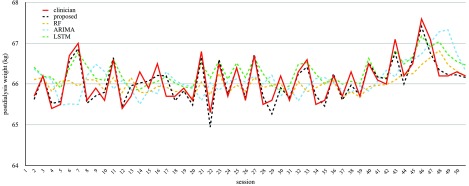
The illustration of different regression algorithms on post-dialysis weight prediction. This result comes from a sample of an HD-stabilized patient. The MAE, MAPE, MSE, and $r^{2}$ from the proposed method are 0.15, 0.22, 0.03, and 0.88, respectively. ARIMA scores are 0.53, 0.8, 0.40, and −0.47. RF scores are 0.35, 0.53, 0.2, and 0.25. LSTM scores are 0.34, 0.52, 0.17, and 0.35.

The mean, standard deviation, and 95% confidence interval for the models discussed in previous sections are summarized in Table 2. The experimental results showed that the proposed model outperformed other methods in terms of mean results regarding all patient groups. The mean of MAE for the HD-stabilized group of the proposed method was 0.17 kg, which is equivalent to 37%, 32%, and 26% improvements compared with ARIMA, RF, and LSTM models, respectively. The MAPE and MSE matrices also confirmed that our proposed method had the best error margin. The $r^{2}$ measurement indicates that the model proposed is the most suitable, as it was 66%, 42%, and 19% better than the ARIMA, RF, and LSTM models, respectively, in HD-stabilized group. The p-value corresponding to the F-statistic of one-way ANOVA [38] in Table 3 suggested that the proposed method was significantly better than one or more methods regarding HD-stabilized group. We then used T-statistic of Bonferroni-Holm [39] in Table 4 to isolate the significance with other methods. The p-value showed that the proposed method was significantly better than ARIMA and RF methods (HD-stabilized group, all metrics). The proposed method showed significantly improved in MAPE (p<0.05) and on the edge of the significance in MAE (p=0.053), MSE (p=0.064), $r^{2}$ (p=0.077) when comparing with LSTM for HD-stabilized group. Figure 6 is an intuitive illustration of the comparisons of different algorithms from one HD-stabilized patient.

**TABLE 2:** Performance Matrices

	Group	Proposed	ARIMA	RF	LSTM
MAE	HD-stabilized	0.17±0.04, [0.15, 0.19]	0.27±0.1, [0.22,.033]	0.25±0.09, [0.2, 0.3]	0.23±0.07, [0.19, 0.26]
	HD-intolerant	0.2±0.05, [0.16, 0.24]	0.35±0.11, [0.25, 0.44]	0.37±0.16, [0.23, 0.52]	0.32±0.12, [0.21, 0.43]
	near-death	0.29±0.16, [0.18, 0.4]	0.48±0.17, [0.36, 0.59]	0.45±0.14, [0.36, 0.55]	0.45±0.14, [0.36, 0.55]
MAPE	HD-stabilized	0.29±0.06, [0.26, 0.33]	0.48±0.18, [0.38, 0.57]	0.44±0.14, [0.36, 0.51]	0.39±0.12, [0.33, 0.46]
	HD-intolerant	0.34±0.09, [0.26, 0.43]	0.62±0.27, [0.37, 0.88]	0.65±0.27, [0.4, 0.9]	0.57±0.26, [0.34, 0.81]
	near-death	0.53±0.33, [0.32, 0.75]	0.89±0.42, [0.61, 1.18]	0.84±0.36, [0.6, 1.08]	0.84±0.34, [0.62, 1.07]
MSE	HD-stabilized	0.05±0.02, [0.04, 0.06]	0.14±0.1, [0.08, 0.19]	0.12±0.07, [0.08, 0.16]	0.1±0.05, [0.07, 0.12]
	HD-intolerant	0.07±0.03, [0.04, 0.1]	0.22±0.14, [0.09, 0.35]	0.25±0.19, [0.07, 0.43]	0.2±0.18, [0.04, 0.38]
	near-death	0.19±0.26, [0.02, 0.37]	0.46±0.31, [0.25, 0.67]	0.45±0.33, [0.23, 0.67]	0.46±0.36, [0.22, 0.7]
$r^{2}$	HD-stabilized	0.78±0.15, [0.71, 0.86]	0.47±0.32, [0.3, 0.65]	0.55±0.28, [0.4, 0.69]	0.63±0.18, [0.54, 0.72]
	HD-intolerant	0.86±0.12, [0.74, 0.98]	0.6±0.38, [0.25, 0.95]	0.64±0.33, [0.34, 0.94]	0.69±0.25, [0.45, 0.92]
	near-death	0.88±0.09, [0.82, 0.95]	0.63±0.26, [0.46, 0.81]	0.66±0.21, [0.52, 0.8]	0.65±0.25, [0.48, 0.81]

^1^ ± means standard deviation value.

^2^ Square bucket shows 95% confidence intervel.

**TABLE 3:** F-Statistic of One-Way ANOVA

Group	MAE p-value	Inference	MAPE p-value	Inference	MSE p-value	Inference	$r^{2}$ p-value	Inference
HD-stabilized	0.0043	** p<0.01	0.002	** p<0.01	0.0034	** p<0.01	0.0042	** p<0.01
HD-intolerant	0.048	* p<0.05	0.084	-	0.1566	-	0.3653	-
near-death	0.0234	* p<0.05	0.097	-	0.1489	-	0.0252	* p<0.05

^1^ - is insignificant.

**TABLE 4:** T-Statistic of Bonferroni-Holm

Group	Method	MAE p-value	Inference	MAPE p-value	Inference	MSE p-value	Inference	$r^{2}$ p-value	Inference
HD-stabilized	ARIMA	0.0017	** p<0.01	0.0008	** p<0.01	0.0014	** p<0.01	0.0018	** p<0.01
	RF	0.0134	* p<0.05	0.008	** p<0.01	0.018	* p<0.05	0.014	* p<0.05
	LSTM	0.0526	-	0.037	* p<0.05	0.064	-	0.0769	-
HD-intolerant	ARIMA	0.0528	-	0.07	-	0.1714	-	0.3131	-
	RF	0.0308	* p<0.05	0.066	-	0.1097	-	0.3282	-
	LSTM	0.056	-	0.233	-	0.1074	-	0.2759	-
near-death	ARIMA	0.0187	* p<0.05	0.0763	-	0.1693	-	0.0265	* p<0.05
	RF	0.0176	* p<0.05	0.0547	-	0.2044	-	0.017	* p<0.05
	LSTM	0.0314	* p<0.05	0.1052	-	0.168	-	0.0248	* p<0.05

^1^ - is insignificant.

## IV. Discussion

The prediction accuracy degraded with worsening patient conditions. The near-death group manifested a substantial accuracy deviation compared with the HD-stabilized group, indicating that the machine learning and statistical-based methods can potentially follow the regularity of the human system. When exceptional medical events happen, the chaos weight adjustment behavior may damage the stability of the model training, which could lead to more significant deviations. From this point of view, the proposed model can behave like a marker to track HD patients’ regularity and exceptional medical events. Naturally, patients with stable life signs may exhibit a better regularity than unstable patients. Since most of the HDs are undertaken at HD centers with nursing staff, it potentially lacks expert knowledge to timely tune or to rise awareness of the risk factors, such as subtle changes in patients’ conditions. The proposed method in this research can supervise and record the weight adjustment behaviors efficiently, such that any operation that drafts off the prediction to a certain threshold will produce a medical warning to alert patients and doctors, and help to buildup personalized HD plans. As aforementioned, the weight prediction model is from the personalized data record, and it avoids the crowd bias of population data and matches the concept of personalized medical care.

## V. Conclusion

This paper proposes a scheme that could process EHR data and establish a DW estimation and supervision model for HD patients. As far as our knowledge goes, this is the first work to have developed a personalized model based on mature DW assessment dataset to monitor DW adjustment behavior. We kept the absolute error under 0.5 kg, and relative error under 1% for most HD-stabilized cases included. This accuracy suggests that this model has great potential for utilization in dialysis centers monitoring the quality of HD automatically. We also observed a more substantial predictive deviations in the HD-intolerant and near-death groups, which indicates that the proposed model should be revised by exceptional medical events to fit situations of clinical instability. The proposed method can be consolidated by further clinical verifications.
